# Fingolimod Therapy in Multiple Sclerosis Leads to the Enrichment of a Subpopulation of Aged NK Cells

**DOI:** 10.1007/s13311-021-01078-7

**Published:** 2021-07-09

**Authors:** Svenja C. Schwichtenberg, Anne Wisgalla, Maria Schroeder-Castagno, Cesar Alvarez-González, Stephan Schlickeiser, Nadja Siebert, Judith Bellmann-Strobl, Klaus-Dieter Wernecke, Friedemann Paul, Jan Dörr, Carmen Infante-Duarte

**Affiliations:** 1 Charité–Universitätsmedizin Berlin, Corporate member of Freie Universität Berlin, Humboldt-Universität Zu Berlin and Berlin Institute of Health, Institute for Medical Immunology, Campus Virchow Klinikum, Augustenburger Platz 1 (Südstr. 2/Föhrer Str. 15), 13353 Berlin, Germany; 2 Charité–Universitätsmedizin Berlin, Corporate member of Freie Universität Berlin, Humboldt-Universität Zu Berlin and Berlin Institute of Health, Institute for “Psychiatrie Und Medizinische Klinik M.S. Psychosomatik,”, Campus Benjamin Franklin, Hindenburgdamm 30, 12203 Berlin, Germany; 3 Charité–Universitätsmedizin Berlin, Corporate member of Freie Universität Berlin, Humboldt-Universität Zu Berlin and Berlin Institute of Health, Neurocure Cluster of Excellence, Campus Mitte, Sauerbruchweg 5, 10117 Berlin, Germany; 4 BIH Center for Regenerative Therapies (BCRT), Charité - Universitätsmedizin Berlin, Campus Virchow Klinikum, Föhrer Str. 15, 13353 Berlin, Germany; 5 Experimental and Clinical Research Center, Max Delbrück Center for Molecular Medicine & Charité - Universitätsmedizin Berlin, Robert-Rössle-Straße 10, 13125 Berlin, Germany; 6 Charité - Universitätsmedizin Berlin and CRO SOSTANA GmbH, Wildensteiner Straße 27, 10318 Berlin, Germany; 7 Current Affiliation: Multiple Sclerosis Center, Oberhavel Kliniken, Marwitzer Straße 91, 16761 Hennigsdorf, Germany

**Keywords:** Natural killer (NK) cells, Fingolimod, Multiple sclerosis (MS), Innate lymphoid cells (ILCs), Sphingosin-1-phosphate receptor (S1PR)

## Abstract

Fingolimod is an approved oral treatment for relapsing–remitting multiple sclerosis (RRMS) that modulates agonistically the sphingosin-1-phosphate receptor (S1PR), inhibiting thereby the egress of lymphocytes from the lymph nodes. In this interventional prospective clinical phase IV trial, we longitudinally investigated the impact of fingolimod on frequencies of NK cell subpopulations by flow cytometry in 17 RRMS patients at baseline and 1, 3, 6, and 12 months after treatment initiation. Clinical outcome was assessed by the Expanded Disability Status Scale (EDSS) and annualized relapse rates (ARR). Over the study period, median EDSS remained stable from month 3 to month 12, and ARR decreased compared to ARR in the 24 months prior treatment. Treatment was paralleled by an increased frequency of circulating NK cells, due primarily to an increase in CD56^dim^CD94^low^ mature NK cells, while the CD56^bright^ fraction and CD127^+^ innate lymphoid cells (ILCs) decreased over time. An unsupervised clustering algorithm further revealed that a particular fraction of NK cells defined by the expression of CD56^dim^CD16^++^KIR^+/−^NKG2A^−^CD94^−^CCR7^+/−^CX_3_CR1^+/−^NKG2C^−^NKG2D^+^NKp46^−^DNAM1^++^CD127^+^ increased during treatment. This specific phenotype might reflect a status of aged, fully differentiated, and less functional NK cells. Our study confirms that fingolimod treatment affects both NK cells and ILC. In addition, our study suggests that treatment leads to the enrichment of a specific NK cell subset characterized by an aged phenotype. This might limit the anti-microbial and anti-tumour NK cell activity in fingolimod-treated patients.

**Supplementary Information:**

The online version contains supplementary material available at 10.1007/s13311-021-01078-7.

## Introduction

Multiple sclerosis (MS) is one of the most frequent chronic neurological diseases in young adults in western countries [1]. MS is traditionally considered a primarily demyelinating autoimmune disease [2–4], in which autoreactive CD4^+^ T cells play a key role in the initiation and maintenance of the chronic inflammation within the central nervous system [5]. This inflammatory process, also supported by B cells and macrophages, eventually leads to demyelination, axonal damage [6] and loss of neurological functions [7].

Fingolimod (Gilenya®) is approved as oral treatment for relapsing–remitting (RR) MS [8, 9]. Its efficacy could be demonstrated in different phase III studies [10–13]. Fingolimod acts by withholding lymphocytes within the lymph nodes through the modulation of the sphingosin-1-phosphate (S1P) receptor [14]. Specifically, fingolimod is an agonist of the S1P_1_ and S1P_3-5_ receptors that are expressed on lymphocytes and regulate cell trafficking [14] as well as vascular barrier function [15, 16], angiogenesis [17], and vascular tone [18]. After its binding, it leads to hyperactivation and subsequently internalization of the receptor, which results in an inhibition of lymphocytic egress from the lymph nodes [19–21] and modulation of the profile of circulating immune cells [22]. S1P receptors are also expressed on innate lymphocytes (ILCs), including NK cells [23–25].

Classically, human NK cells are subdivided according to their CD56 expression into CD56^bright^ and CD56^dim^ NK cells [26]. Approximately 90% of the peripheral NK cells in circulation are CD56^dim^, whereas CD56^bright^ NK cells are more abundant in secondary lymphoid tissues and within the cerebrospinal fluid [27–29].

To date, both beneficial and deleterious roles of NK cells have been proposed in MS [30–33]. Reports of the last four decades indicate that MS development is associated with a deficient NK cell activity [34–41]. However, other studies showed a relationship between NK cell action and central nervous system pathology [42–47], indicating that the effect of NK cells on MS may depend on the NK cell type or mode of action.

Treatment-related enrichment of a particular NK cell subset, mostly CD56^bright^, or induction of NK cell activation has been associated with the therapeutic success of numerous MS drugs including interferon-beta, daclizumab, mitoxantrone, glatiramer acetate, or alemtuzumab [36, 48–59].

Effects of fingolimod on NK cells have also been extensively investigated [22, 52, 60–70]. Apart from one study that did not observe any significant effect of fingolimod on NK cells [22], all other cross-sectional studies have shown a relative increase of NK cells compared with other lymphocyte populations. These studies also revealed a decrease of CD56^bright^ in comparison to CD56^dim^ in fingolimod-treated patients compared with an untreated cohort, healthy controls, or patients treated with other medications [60, 61, 64]. Interestingly, although proportions but not absolute numbers of NK cells and CD56^dim^ were affected by fingolimod treatment, significant reduction in CD56^bright^ counts was observed [63, 67, 68, 71]. Longitudinal intra-individual studies corroborated the altered frequencies of NK cells and CD56^bright^ and CD56^dim^ [62, 64, 65, 68, 69]. However, the specific immune profiles of these enriched or depleted subpopulations remain largely undetermined.

In a more recent publication, Eken et al. [25] showed a decrease of all types of ILCs in the peripheral blood of fingolimod-treated patients comparing off and on treatment.

It has been demonstrated that the expression of S1P receptors increases during NK cell maturation [24]. Thus, different NK cell subtypes may respond differently to S1P-receptor agonist such as fingolimod.

Therefore, in this study, we aimed to investigate longitudinally the effects of fingolimod on NK cell homeostasis and maturation/differentiation to define more comprehensively the profile of NK cells enriched under fingolimod therapy. We hypothesized that fingolimod specifically targets certain NK cell subsets and that this effect may be associated with therapeutic benefits.

## Methods

### Study Design

A post-authorizational investigator-driven, interventional, prospective, open-label, baseline-to-treatment study was designed to evaluate the effects of fingolimod treatment on NK cells in RRMS patients. The study (Eudra-CT:2012–000,411-91; NCT 01790269) was approved by the responsible ethics committee (Approval ID: 12/9543-EK) and regulative authorities and was conducted in accordance with the Declaration of Helsinki, the guidelines of the International Conference on Harmonization of Good Clinical Practice, and the applicable German laws. All participants gave informed written consent. Patients were screened and enrolled at the Experimental and Clinical Research Center and the Neurocure Research Center at Charité-Universitätsmedizin Berlin. The main inclusion criteria were RRMS according to the 2010 McDonald criteria [72] with an Expanded Disability Status Scale (EDSS) ≤ 6.0 [73], age 18–64 years, an indication for on-label treatment with fingolimod, and absence of relapse for 30 days prior to screening. Exclusion criteria were mainly related to the contraindications of fingolimod as indicated in the label. An additional exclusion criterion was the intake of other disease modifying drugs within 6 months (regarding mitoxantrone, azathioprine, or any other immunosuppressive drug except prednisolone) or 3 months (natalizumab) prior to baseline. Detailed inclusion and exclusion criteria are provided as supplementary Table 1.

Effects of fingolimod on NK cell maturation, differentiation, and activation were investigated longitudinally (baseline vs. treatment) by flow cytometry. Blood samples were obtained at baseline visit (visit 0) and 1 month (visit 1), 3 months (visit 2), 6 months (visit 3), and 12 months (visit 4) after treatment initiation. The primary endpoint was the degree of NK cell maturation, defined as the ratio of immature NK cells/total NK cells (percentage) before fingolimod treatment and after 12 months of treatment. Secondary endpoints included NK cell frequency, percentage of immature NK cells/total NK cells, and degree of NK cell activation and maturation at all time points. Clinical secondary endpoints included the number of relapses, the disability profile (determined by the EDSS [73]), and side effects determined by the numbers of adverse events and infections.

### Study Drug Application

Fingolimod in 0.5mg capsules was administered orally once daily for a period of 12 months. The treatment phase began at the baseline visit within 4 weeks after the screening visit. Treatment exactly complied with the approved label, dose, application, frequency, and safety monitoring. All regular study visits coincided with the regular treatment monitoring visits, but included larger samples of venous blood (ca. 40 ml).

### Sample Collection and Flow Cytometry

Heparinized peripheral blood samples were collected during study visits. Peripheral blood mononuclear cells (PBMCs) were isolated by density gradient centrifugation (Biochrom GmbH, Germany) according to the manufacturer’s instructions as described previously [56], and were then cryopreserved in liquid nitrogen for later analysis. All samples of the same patient (different time points) were processed and analyzed simultaneously.

For the characterization of NK cell subpopulations, defrozen PBMCs were washed and incubated with human Fc fragments (Miltenyi Biotec, Germany) to block unspecific antibody binding, followed by the incubation with anti-CCR7-BV421, anti-NKG2C-AlexaFlour488, anti-DNAM-1/CD226-PerCP/Cy5.5, anti-CD158a/h-PE, anti-CD56-PE/Dazzle594, anti-NKG2A/CD159a-PE/Cy7, anti-CD94-APC, anti-Lin( CD3, CD19, CD14, CD20)-Alexa700, anti-CD16-APCfire750, anti-NKG2D-BV510, anti-CX_3_CR1-BV605, anti-NKp46-BV650 and anti-CD127-BV785. Viable cells were identified with “LIVE/DEAD™ Fixable Dead Cell Stain Kits “ (Thermofisher). Data were acquired at Cytoflex LX flow cytometer (Beckman Coulter).

### Clinical Data Collection and Analysis

As fingolimod shows a steady-state blood concentration only after approximately 2 months of treatment [74], the first 3 months of treatment were not considered when assessing clinical effects. Effect of treatment on relapse rates was therefore evaluated by comparing the annualized rate of relapses during treatment (from month 3 to 12) to the annualized rate in the 24 months prior study.

Relapses were defined as either the occurrence of new neurological symptoms or the recurrence or worsening of previously existent symptoms/signs at least 30 days after the beginning of a previous demyelinating event. The symptoms/signs had to last at least 24 h and had to occur independently of fever or acute infections. The course of MS-related disability determined by EDSS [73] was evaluated by comparing the EDSS from month 3 and month 12.

Treatment response was defined as the absence of EDSS increase from month 3 to month 12 and decrease in ARR when comparing the ARR from month 3 to month 12 on-treatment with the 24 months pre-treatment ARR. Non-response was defined as EDSS increase from month 3 to month 12 and/or stable ARR or increase in ARR when comparing the ARR from month 3 to month 12 on-treatment with the 24-month pre-treatment ARR. If no relapse occurred during the study and 24 months prior to study and the EDSS did not increase from moth 3 to month 12, the patient was considered a responder.

### Flow Cytometry Data Analysis and flowSOM-Based Unsupervised Analysis

**Fig. 1:**
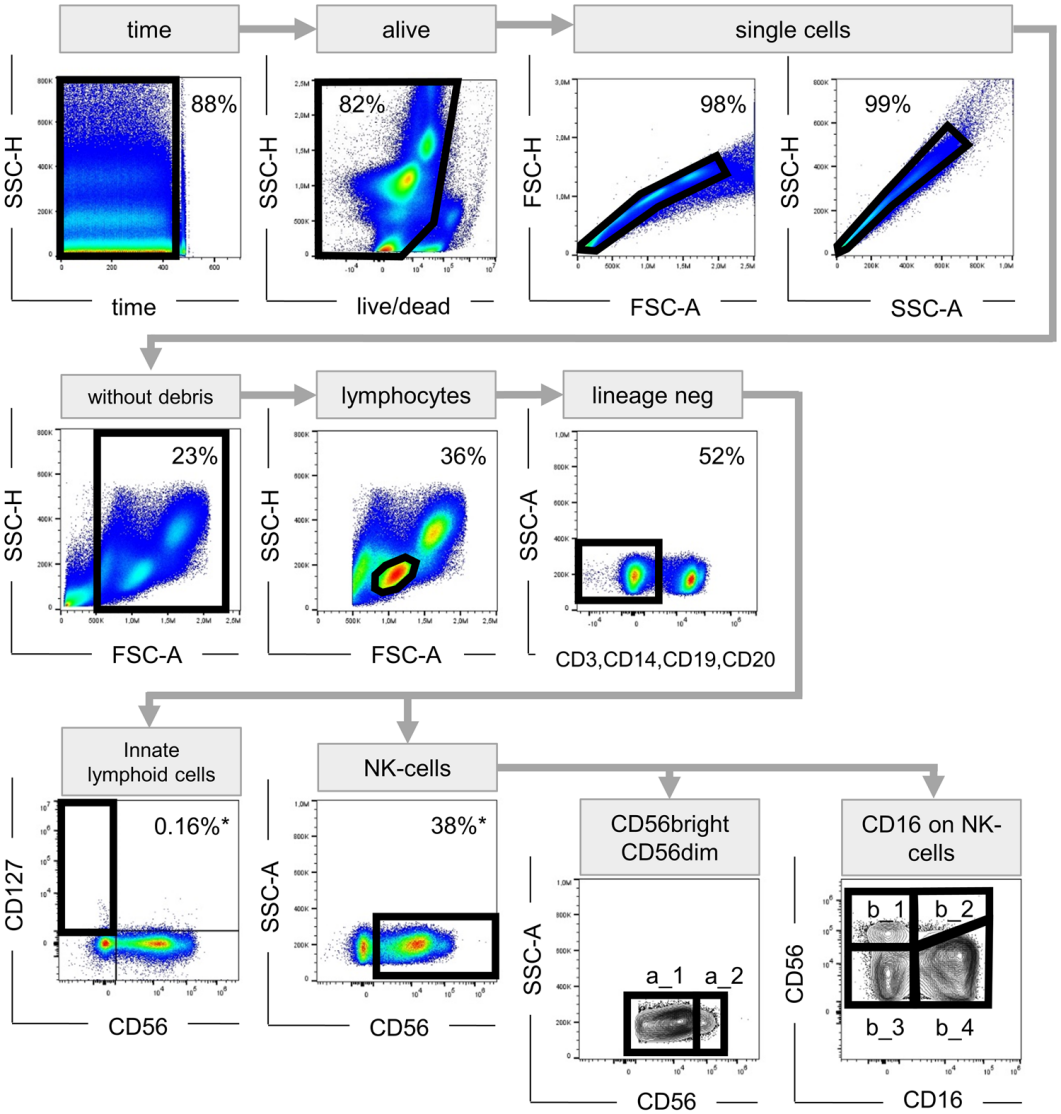
Gating strategy. Artefacts caused by poor flow were eliminated in a time vs. scatter plot; afterwards, dead cells as well as cell clumps or doublets were excluded; to disregard cell debris, only events > 500,000 on the FSC-A scale were included in further analysis; lymphocyte population was determined by its characteristic morphology in FSC-A vs. SSC-H plot; T, B, NKT cells and monocytes were excluded from the analysis by staining for CD3, CD19, CD14, or CD20, respectively. ILCs were described as lineage-negative, CD56^−^CD127^+^ cells. NK cells were defined as lineage-negative CD56^+^ cells. The CD56^+^ cells were further discriminated in CD56^dim^ (a_1) and CD56^bright^ (a_2) cells. The expression of the other markers of interest (CCR7, NKG2C, DNAM-1, CD158a/h, NKG2A, CD94, NKG2D, CX_3_CR, NKp46) were analysed considering both, all NK cells and the subgroups (CD56^dim^ and CD56^bright^). NK cells were also subcategorized according to CD16 expression in CD56^bright^CD16^−^ (b_1), CD56^bright^CD16^+^ (b_2), CD56^dim^CD16^−^ (b_3), and CD56^dim^CD16^+^ (b_4). FSC forward scatter, SSC side scatter, -H pulse height, -A pulse area

The flow cytometry data were first analysed unblinded using FlowJo software 10.4 (TreeStar). Thereafter, a multidimensional unsupervised analysis was conducted using Cytobank (www.cytobank.org). Fluorescent minus one (FMO) stainings were used as controls. The applied gating strategy is shown in Fig. 1. From the 84 acquired data points, 13 measurements were excluded during the analysis process due to poor sample quality (very high number of dead cells or debris) or defective acquisition. These samples were excluded before the initiation of the analysis.

Expression of a given marker was determined either by percentage of fluorescence positive cells or by using the mean fluorescence intensity (MFI). In the latter case, a change in the MFI (Δ MFI) was calculated by subtracting the MFI of the FMO-control from the FMI of the specific marker.

In addition, to avoid biased analysis based in pre-determined populations, we performed a multidimensional unsupervised analysis using the FlowSOM algorithm [75]. For that, all compensated files were transferred to the Cytobank and scaled in this program. Events (cells) from all included patients and all visits were assigned to a self-organizing map. Events with similar properties were aggregated in one of different 81 clusters. A minimal spanning tree visualized similar clusters in the same branch of this tree. Clusters with similar properties were then further integrated within 15 final metaclusters (MCs). To assess effects of treatment on NK cell clusters, MC percentage at baseline visit and after 12 months of therapy were compared. Next, a qualitative analysis of differences of the MCs in responders and non-responders was conducted.

### Statistical Analysis

Data of primary and secondary endpoints and the MCs were analyzed using an exploratory approach and descriptive statistics (frequency of parameters, median). To assess the primary endpoint, status of NK cell maturation (ratio immature NK cells per total NK cells as a percentage) before treatment and after 12 months of treatment was compared. Secondary immunological endpoints (NK cell frequency, ratio immature NK cells per total NK cells, as well as the frequency of NK cell activation and functional markers) were evaluated for the whole treatment period. Additionally, the frequency of the MCs from the unsupervised clustering analysis was evaluated for all time points. Non-parametric (pairwise exact Wilcoxon) analysis was performed to compare (univariately) immune subsets before treatment and after 12 months of treatment. In order to test for systematic changes over time, markers of special interest with a significant Wilcoxon result were included in a nonparametric analysis of longitudinal data (nonparametric MANOVA) [76]. Univariate post hoc Wilcoxon tests after global testing were used to check for significant differences in certain clinically interesting time points.

Generalized estimating equations (GEE) were applied to test for potential associations between clinical parameters and changes in immunological endpoints over time. Different GEE models were examined with number of infections, number of relapses, and frequency of CD56^bright^, MC4, and CD56^dim^CD94^low^ cells as independent influencing factors. Based on our previous experiences in neurology [77], we used an autoregressive model of 1st order for the correlation matrix.

Tests should be seen as exploratory data analysis. Therefore, all p-values have to be understood as exploratory ones. For that reason, no adjustments for multiple testing were conducted. Statistical significance was defined as p < 0.05. Calculations were performed using IBM© SPSS© Statistics, Version 25, © Copyright 1989, 2016 SPSS Inc., an IBM Company and the R Project for Statistical Computing, Version 3.4.0 (2017–04-21). Figures were generated using GraphPad prism 8.0.0.

## Results

### Demographic and Clinical Data

Twenty-one patients were screened and included in this study. Three patients terminated fingolimod treatment early due to a significant elevation of liver enzymes (2 patients) or severe lymphocytopenia (1 patient) and therefore dropped out. Another patient developed severe lymphocytopenia (grade 4 toxicity) before the last visit; for this patient, the data of only 4 time points were analyzed. Data of another patient were removed from the analysis because of the poor quality of the frozen blood samples.

The final analysis included therefore 16 patients treated daily for 12 months and one patient treated for 10 months. Demographic baseline data are displayed in Table 1. Females accounted for 65% of the cohort; the mean age at screening was 41 years; the mean MS duration was 10 years. The median EDSS score at treatment start was 2.0. Fifteen (88%) of the 17 patients received previous treatment with other disease-modifying drugs. Based on the response criteria described in “Methods,” seven patients (41%; 2 males, 5 females) were classified as non-responders.

**Table 1:** Demographic characteristics

	n = 17
Age (in years, mean ± SD)	40.8 ± 10.0
Females (n (%))	11 (64.7)
Interval from MS onset to baseline (in years, mean ± SD)	10.1 ± 6.7
Annualized relapse rate (ARR) 24 months before baseline	0.68
EDSS score at baseline (median (range))	2.0 (0.0–6.0)

*SD* standard deviation

From the patients included in the analysis, 24% (n = 4) suffered from one or more relapses during the study. A total of 18% (n = 3) showed an increase in the ARR calculated from month 3 to month 12 on-treatment compared with the 24-month pre-treatment ARR. Median EDSS remained stable from visit 2 (third month of treatment) to the last visit after 12 months of treatment. In 35% (n = 6), an EDSS increase was observed. Thus, relapses and/or EDSS increase were observed in seven patients that for hypothesis generating analyses were considered as non-responders.

Serious adverse events were reported in three patients, namely elevated liver enzymes, herpes zoster, and urosepsis with subsequent aggravation of MS-symptoms. No serious adverse events resulted in hospitalization, death, or a permanent disability. Fourteen infection-related adverse events were recorded, seven in the responder group, affecting four patients, and seven in the non-responders, that affected five out of the seven patients. Therefore, as shown in Table 2, the number of patients affected by infections was higher in the non-responder group (71%) than in the responder group (40%).

**Table 2:** Clinical outcomes

Relapses	All patients (n = 17)	Responder (n = 10)	Non-responder (n = 7)
Patients with confirmed relapse from visit 2 to treatment termination (n (%))	4 (23.5)	0 (0.0)	4 (57.1)
1 relapse (n(%))	2 (11.8)	0 (0.0)	2 (28.6)
2 relapses (n(%))	2 (11.8)	0 (0.0)	2 (28.6)
Annualized relapse rate (ARR)	0.47	0	1.14
**EDSS**			
Patients with EDSS increase from visit 2 to treatment termination (n (%))	6 (35.3)	0 (0.0)	6 (85.7)
Change from visit 2 to visit 4 (median (range))	0.0 (− 1.5–1.5)	− 0.5 (− 1.5–0)	0.5 (0.0–1.5)
**Infections**			
Patients with infections from visit 2 to treatment termination (n (%))	9 (53.0)	4 (40.0)	5 (71.4)
Number of infections from visit 2 to treatment termination	14	7	7

### Effects of Fingolimod on Immature/Mature NK Cell Fractions

NK cell markers were analysed according to the gating strategy shown in Fig. 1. The expression of maturation markers was analysed considering all NK cells or the CD56^dim^ and CD56^bright^ fractions separately as displayed in Fig. 1. The following statistical analyses should be seen as exploratory data analysis; therefore, all p-values are to be understood as exploratory ones.

**Fig. 2:**
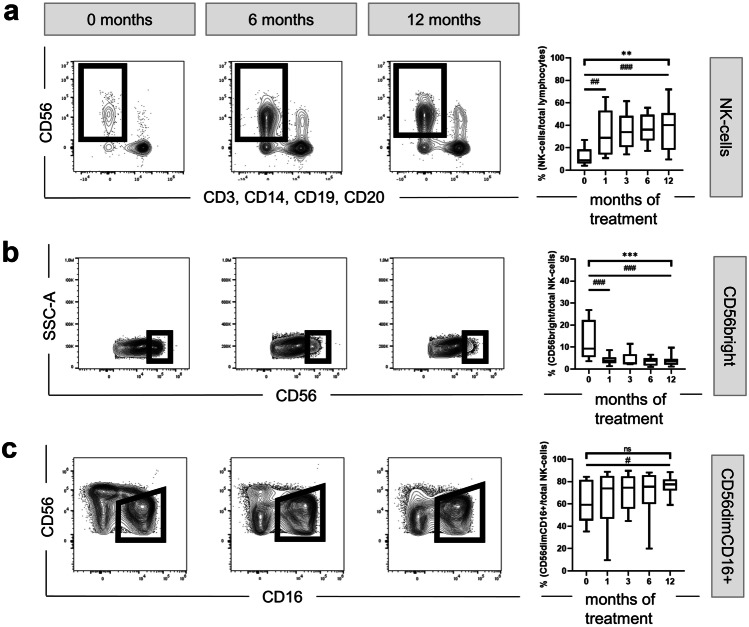
Effect of fingolimod therapy on the expression of CD56 and CD16. Representative FACS plots displaying fingolimod-induced changes over time in several NK cell populations are depicted on the left-side. Box-and-whisker plots show the corresponding quantification for all patients at all timepoints. **a** Analysis of all NK cells over time. **b** Analysis focusing on CD56^bright^. **c** Analysis of NK cells according to their CD56 and CD16 expression: The CD56^dim^CD16^+^ population is framed. The corresponding box-and-whisker plot shows changes overtime affecting the CD56^dim^CD16^+^. MANOVA *p ≤ 0.05; **p ≤ 0.01; ***p ≤ 0.001; exact Wilcoxon test #p ≤ 0.05; ##p ≤ 0.01; ###p ≤ 0.001; SSC side scatter, -A pulse area

Figure 2A shows that the frequency of circulating CD56^+^ NK cells increased during treatment (MANOVA p = 0.003), from 8.74% before treatment to 28.90% after 1 month (Wilcoxon test p = 0.005) up to 40.05% at month 12 (Wilcoxon test p < 0.001).

Further, we observed a fingolimod-associated reduction of CD56^bright^ NK cells over time (MANOVA p = 0.0000009) from 9.30% at baseline to 3.83% after 1 month (Wilcoxon test p < 0.001) and 3.56% after 12 months of treatment (Wilcoxon test p < 0.001) (Fig. 2B). However, as shown in Fig. 2C, within the CD56^dim^ population only, the fraction of circulating CD16^+^ CD56^dim^ NK cells increased during treatment from baseline median 59.30 to 77.43% after 12 months of fingolimod intake (Wilcoxon test p = 0.042). Additionally, the analysis of ILCs, defined as CD56^−^CD127^+^ cells, revealed a mild decrease over the treatment period from 0.17 to 0.12% (Wilcoxon test p = 0.01) (data not shown).

**Fig. 3:**
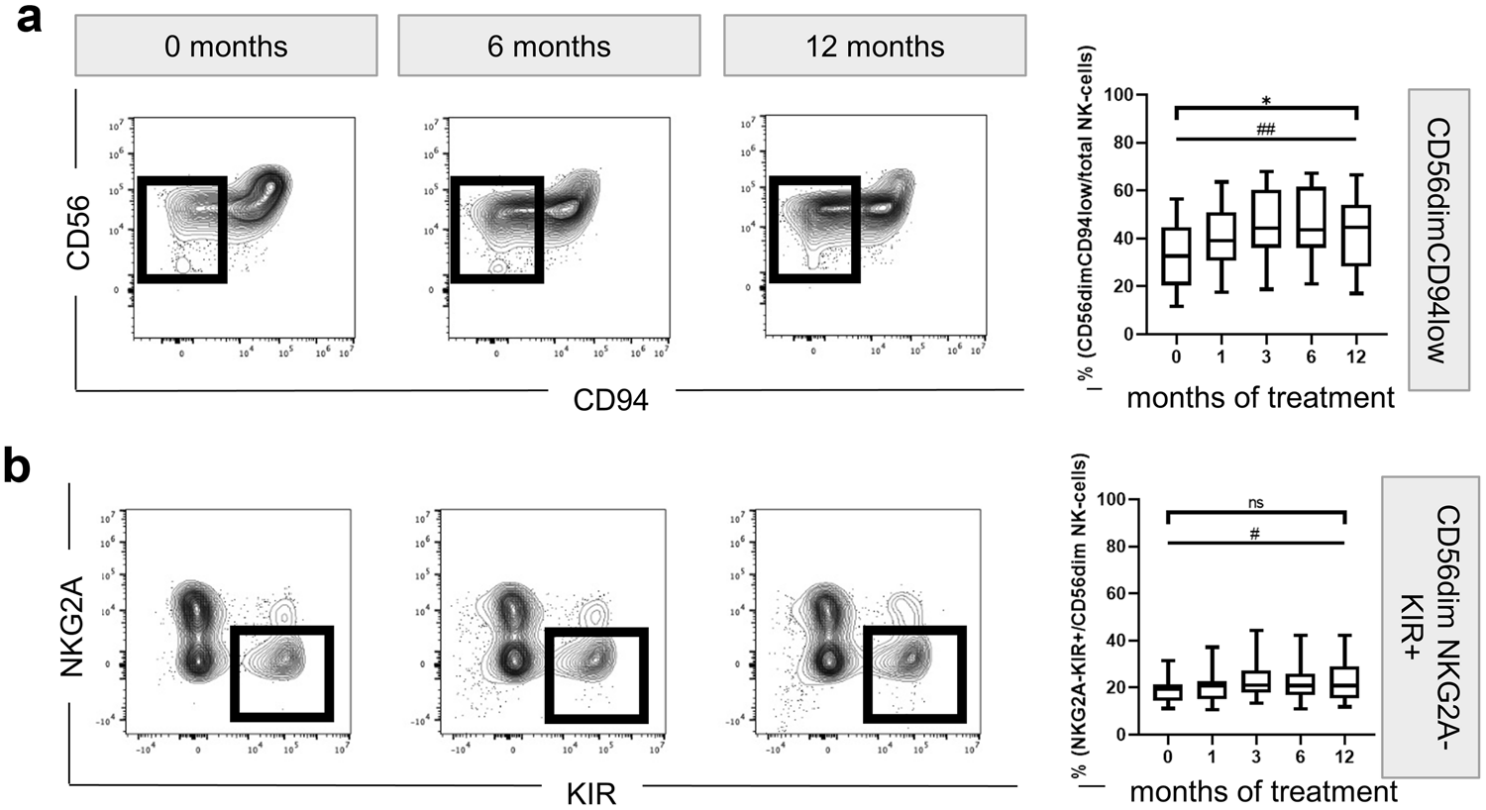
Effect of fingolimod therapy on NK cell maturation markers. Representative FACS plots and the corresponding quantification are depicted on the left- and right-side, respectively. **a** Frequency of CD56^dim^CD94^low^ cells from the CD56 fraction. **b** Quantification of the NKG2A^−^KIR^+^ cells from the CD56^dim^ fraction. MANOVA *p ≤ 0.05; **p ≤ 0.01; ***p ≤ 0.001; exact Wilcoxon test #p ≤ 0.05; ##p ≤ 0.01; ### p ≤ 0.001

Further, we focused on the fully mature fractions, that are CD94^low^ [78] and express KIR, but no NKG2A [79, 80]. We observed an increase of fully mature CD56^dim^CD94^low^ over time (MANOVA p = 0.014) (Fig. 3A) from 32.65% at baseline to 44.60% after 12 months of treatment (Wilcoxon test p = 0.008). In the same line, NKG2A^−^KIR^+^ CD56^dim^ NK cells increased slightly from 19.30% at treatment initiation to 20.85% after 12 months of fingolimod intake (Wilcoxon test p = 0.013) (Fig. 3B).

Thus, the proportion of highly mature NK cells increased after 1 month of treatment and remained elevated during the treatment period of 12 months (overview supplementary Table 2).

### Activation and Chemotactic Capacity of NK Cells Under Fingolimod Treatment

To evaluate the proportion of NK cells with activating or migration-mediating receptors during the treatment with fingolimod, we examined the expression of the chemokine receptors CCR7 and CX_3_CR1, as well as the inhibition marker NKG2A and the activation markers NKp46, DNAM-1, NKG2D, and NKG2C.

**Fig. 4:**
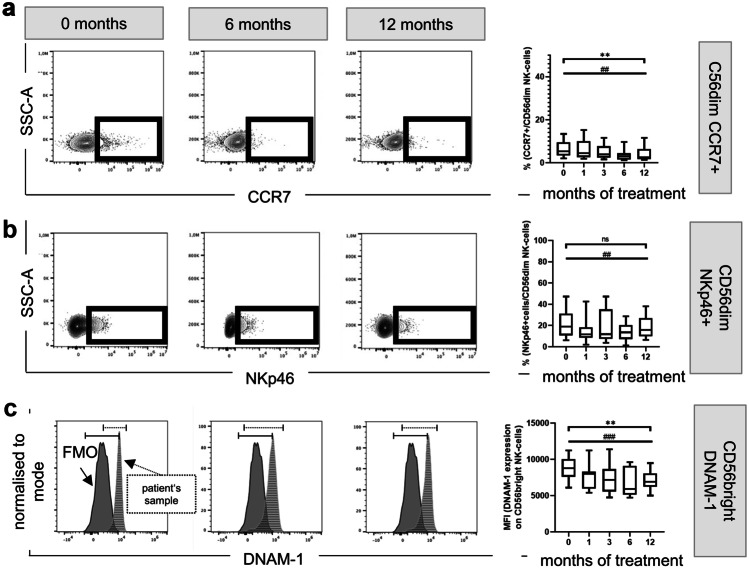
Effect of fingolimod therapy on NK cell activation and chemotactic responses. Representative FACS plots and a histogram displaying fingolimod-induced changes over time of CCR7 (**a**), NKp46 (**b**), and DNAM-1 (**c**) are depicted on the left-side. Box-and-whisker plots on the right-side show the corresponding quantification for all patients at all time points. MANOVA *p ≤ 0.05; **p ≤ 0.01; ***p ≤ 0.001; exact Wilcoxon test #p ≤ 0.05; ##p ≤ 0.01; ###p ≤ 0.001; SSC side scatter, -A pulse area, FMO fluorescence minus one, MFI median fluorescence intensity

Fingolimod treated patients showed a decreased proportion of both CCR7^+^ and CX_3_CR1^+^ CD56^dim^ NK cells in peripheral blood. As shown in Fig. 4A, the CCR7 expressing CD56^dim^ NK cells decreased from treatment initiation to the later visits (MANOVA p = 0.0032). The fraction of CCR7^+^ CD56^dim^ NK cells decreased from 5.30% at baseline to 2.46% after 12 months of treatment (Wilcoxon test p = 0.007) (Fig. 4A). We observed a trend in the decrease in fluorescence intensity of CX_3_CR1 on CD56^dim^ NK cells (p = 0.056) (supplementary Fig. 1B). No change in CCR7 or CX_3_CR1 was observed in CD56^bright^ NK cells (supplementary Fig. 1A, 1B).

Furthermore, the proportion of the CD94^+^NKG2A^+^ NK cells was reduced from 51.60 to 47.60% at the 12-month visit (Wilcoxon test p = 0.004). The frequency of CD94^+^NKG2A^+^ cells also decreased in both CD56^dim^ and CD56^bright^ NK cells after 1 month of treatment and remained low during the entire treatment period (supplementary Fig. 1C).

Next, we analysed changes in the percentage of NK cell activation receptors. While the ratio of NKp46^+^ and DNAM-1^+^ NK cell subpopulations was consistently reduced after 1 month of fingolimod intake, we observed no significant changes in NKG2D^+^ and CD94^+^NKG2C^+^ NK cells (supplementary Fig. 1D, 1E, 1F, 1G). The proportion of NKp46 expression only decreased in CD56^dim^ NK cells, while CD56^bright^ NK cells were not affected (supplementary Fig. 1D). While the decrease from baseline (median frequency: 18.90%) to 12 months of treatment (median frequency: 15.80%) was significant (Wilcoxon test p = 0.007) in the CD56^dim^NKp46^+^ cells, no alteration over the entire treatment period was observed (Fig. 4B).

On the other hand, the ratio of DNAM-1 expressing CD56^bright^ decreased during the observation period (MANOVA p = 0.0036) (Fig. 4C). In line with these findings, we could also evaluate a significant decrease in the proportion of DNAM-1 in CD56^dim^ NK cells comparing baseline to 12 months (Wilcoxon test p = 0.035) (supplementary Fig. 1E).

Thus, fingolimod may affect NK cell function by promoting a reduced fraction of activation receptors expressing NK cells. This was observed for the entire NK cell population and also for the CD56^bright^ and CD56^dim^ fractions (overview supplementary Table 2).

### Unsupervised Multidimensional Analysis of the Effect of Fingolimod on NK Cell Subpopulations

**Fig. 5:**
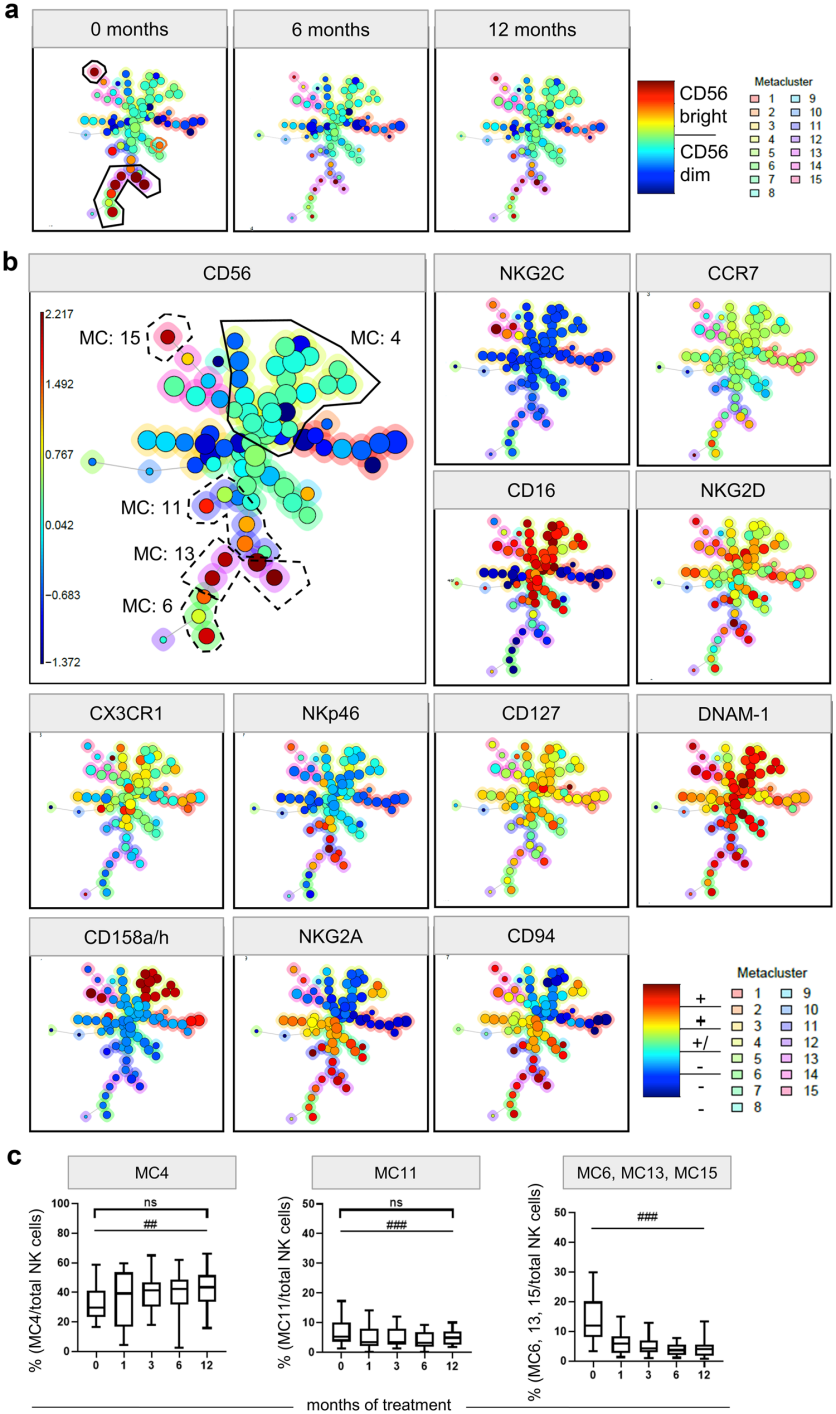
FlowSOM unsupervised clustering of CD56^+^ NK cells in fingolimod treated patients. The FlowSOM clustering was performed by analysing the expression of CCR7, NKG2C, DNAM-1, 158a/h, CD56, NKG2A, CD94, CD16, NKG2D, CX_3_CR1, NKp46, and CD127 on each cell for all samples. Cells with similar characteristics are merged into one cluster, shown as individual nodes in the minimum-spanning tree (MST). Clusters with similar conditions are pictured close to each other. The median fluorescence intensity of each marker is visualized by the colours; red represents high expression, while blue represents low marker expression. The colour around each cluster shows the MC this cluster belongs to. The cluster size represents the number of cells that integrates this cluster. **a** CD56 expression on NK cells at treatment start (0 months) and after 6 and 12 months of treatment. The CD56^bright^ clusters are framed. CD56^bright^ clusters decreased in size, while CD56^dim^ cluster increased from treatment start to 6 and 12 months of treatment. **b** Expression of different markers. The MCs with significant changes are framed. The continuous line encloses the MC that increased, and the dashed line is around the MCs that decreased over the treatment period. **c** Box-and-whisker plot of the percentage of MC4 per total NK cells, MC11 per total NK cells, and the sum of MC6, 13 and 15 (all MC are CD56^bright^) per total NK cells of all included patients at treatment start (0 months) and 1, 3, 6, and 12 months after treatment. MANOVA *p ≤ 0.05; **p ≤ 0.01; ***p ≤ 0.001; exact Wilcoxon test #p ≤ 0.05; ##p ≤ 0.01; ###p ≤ 0.001; MC metacluster

To investigate whether a so far undescribed fraction of circulating NK cells increases in fingolimod treated patients, the high-dimensional data set was further explored by means of the unsupervised clustering algorithm FlowSOM. FlowSOM clusters and reduces the dimensionality by displaying the data of all markers and patients in one self-organized map (SOM) [75]. From 83 flow cytometry files of all patients included in this study, which were manually pre-gated for the CD56^+^ NK cell (as shown in Fig. 1), a minimum-spanning tree (MST) was generated. Clusters with cells characterized by similar phenotypes are depicted in the same branch of the tree. Data was merged into a total of 81 clusters and further aggregated into 15 MC. Figure 5A illustrates the MST by displaying the median CD56 signal intensity for each cluster node coded by colour. Cluster size reflects the number of events included in it. Here, CD56^bright^ NK cell cluster (black frame) can be easily identified and showed a decrease over time (Fig. 5A).

The comparison of all MC revealed that three inter-related MC (MC6, 13, and 15) decreased significantly comparing baseline and 12 months of treatment (Wilcoxon test p = 0.001). As shown in Fig. 5B, all three MCs are characterized by a high expression of CD56^bright^ (orange or red cluster nodes). While the baseline-frequency of MC6, MC13, and MC15 was 11.84%, it decreased to 5.90% after 1 month and further decreased to 4.00% after 12 months of treatment (Fig. 5C). Unsupervised analyses confirmed our previous results that the proportion of NK cell with bright CD56 expression decreased during treatment.

Further, we identified a significant increase in the frequency of MC4, containing CD56^dim^ expressing NK cells and a decrease in the frequency of MC11, containing NK cells with different levels of CD56 expression (Fig. 5B). The MC4 fraction expanded significantly from a baseline median frequency of 29.59 to 43.45% after 12 months of therapy (Wilcoxon test p = 0.01), while the MC11 fraction slightly decreased from median 5.34 to 4.90% (Wilcoxon test p < 0.001) (Fig. 5C).

**Fig. 6:**
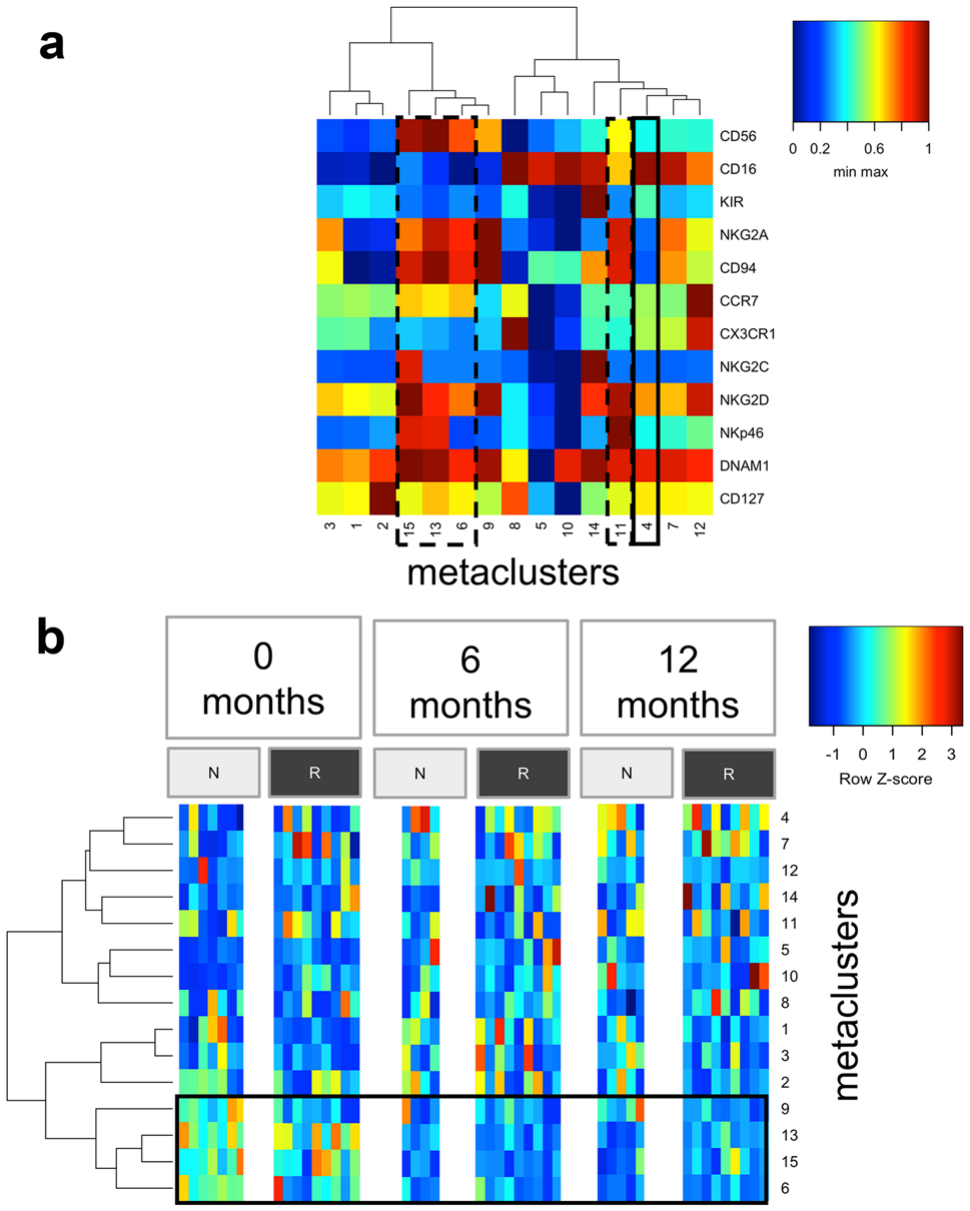
Heatmaps of the MC. **A** Heatmap of all MCs on the basis of the median expression of 12 markers with a colour spectrum scaled per marker from minimum to maximum fluorescence intensity. The continuous black line encloses the MC4 that increased significantly over time; dashed black lines define the MCs that decreased significantly during the treatment (MC 6, 11, 13, 15). **B** Direct comparison of MC frequencies in responders (“R,” dark grey) and non-responders (“N,” light grey) in a heatmap for baseline and the visits after 6 and 12 months of treatment. Each column represents the MC frequencies of one patient for one specific timepoint during the therapy. To improve the comparability between the different MCs and accounting for subject-specific ranges, we showed the frequencies with a row z score normalization (per sample across all MCs). CD56^bright^ MCs framed. MC metacluster

To define the phenotype of cells within a MC, median expression of all analysed markers with a colour spectrum scaled per marker from minimum to maximum fluorescence intensity are depicted in Fig. 6A. While MC4 cells are CD16^++^KIR^+/−^NKG2A^−^CD94^−^CCR7^+/−^CX_3_CR1^+/−^NKG2C^−^NKG2D^+^NKp46^−^DNAM-1^++^CD127^+^, NK cells of the MC11 are CD16^+^KIR^−^NKG2A^++^CD94^++^CCR7^+/−^CX_3_CR1^+/−^NKG2C^−^NKG2D^++^NKp46^++^DNAM1^++^ CD127^+/−^. Thus, in comparison to MC4, MC11-NK cells express lower levels of CD16, KIR, and CD127 and higher levels of NKG2A, CD94, NKG2D, and NKp46 and could be considered as an intermediate mature NK cell population.

Next, data from patients considered as responders and non-responders were mapped to the MST separately. The frequency of the different MCs depicted with a colour scale at baseline, as well as after 6 and 12 months of treatment, is represented in Fig. 6B. We observed a higher frequency of the CD56^bright^ MCs (MC6, 9, 13, 15) and the CD127^+^ MC2 in the non-responders compared to the responders at baseline visit. After treatment initiation, these MCs seem to decrease in both non-responders and responders and converge to same levels.

### Longitudinal Association of Fingolimod-Induced Changes in NK Cell Subpopulations with Clinical Outcome Parameters

Further, we performed a hypothesis-driven analysis, in which three different NK cell subpopulations were investigated as independent variable for associations with clinical endpoints (number of infections, number of relapses, number of adverse events, EDSS, and response) within the 12 months of treatment. The independent variable were CD56^bright^ cells, a regulatory, low cytotoxic NK cell subpopulation [81], CD56^dim^CD94^low^ cells, a mature subpopulation with low regulatory, but high cytotoxic capacity [78] and MC4 cluster, a yet undescribed NK cell cluster that showed a significant fingolimod-induced increase in the previous unsupervised clustering analysis.

Generalized estimating equation (GEE) analysis revealed interesting initial data. We measured an inverted correlation between changes in CD56^dim^CD94^low^ and number of infection (Table 3), indicating that an increase in proportion of one percent of this population correlates with a decreased risk for an infections by 3.7% (odds ratio = 0.963; 95% confidence interval = 0.939–0.988, p = 0.004). In addition, a decrease of the CD56^bright^ fraction correlated with a relapse risk increase (decrease by 1% was associated with an increase of the risk for a relapse by 25.9%, odds ratio = 0.741; 95% confidence interval = 0.556–0.998, p = 0.041)]. No significant correlations between the three NK cell populations and number of AEs, EDSS, or response were found.

**Table 3:** Longitudinal association of clinical outcome parameters and fingolimod-related NK cell profile

		Coefficient (95% CI)	p-value	Odds ratio (95% CI)
Dependent variable: number of infections				
Independent variable:	Change in CD56^bright^	−0.080 (−0.169–0.008)	0.073	0.923 (0.845–1.008)
	Change in MC4	−0.010 (−0.040–0.020)	0.503	0.990 (0.960–1.020)
	Change in CD56^dim^CD94^low^	** −0.037 (−0.063 to −0.012)**	**0.004**	**0.963 (0.939–0.988)**
Dependent variable: number of relapses				
Independent variable:	Change in CD56^bright^	** −0.300 (−0.587–0.012)**	**0.041**	**0.741 (0.556–0.988)**
	Change in MC4	0.037 (−0.005–0.080)	0.085	1.038 (0.995–1.083)
	Change in CD56^dim^CD94^low^	0.043 (−0.007–0.094)	0.093	1.044 (0.993–1.099)

Results for two separate multivariate generalized estimating equation (GEE) analyses, using the “number of infection” or “number of relapses” as dependent variables, respectively. As independent parameters CD56^bright^, MC4 and CD56^dim^CD94^low^ were used in both models. Analyses were conducted to check for prediction of the clinical parameters by alteration of the immune populations. Values in bold indicate significant correlations

To summarize, an increase in the fraction of CD56^dim^CD94^low^ and CD56^bright^ NK cell populations was associated with a reduced number of infections and with a reduced number of relapses, respectively.

## Discussion

This pilot study aimed to determine the effects of 12 months of fingolimod treatment on frequency and phenotype of circulating NK cells in a cohort of MS patients in the context of an exploratory flow cytometry analysis.

We observed an increase of NK cell fraction within the lymphocytes over the treatment period (Fig. 2A), which is consistent with previous reports [60–62, 64, 65, 68, 69]. Hjorth et al. [69] demonstrated a decrease in number of circulating lymphocytes in fingolimod treated patients, while NK cell numbers were not affected. NK cells, contrary to T and B cells, only express low levels of the major fingolimod target S1P_1_ receptor [23, 24]. Thus, the increase of NK cell frequency observed in our study may result from the decreased number of circulating adaptive lymphocytes that, in contrast to NK cells, are broadly sequestered within the secondary lymphoid tissue during fingolimod treatment [82].

Moreover, our study confirmed that fingolimod reduces the proportion of circulating ILCs [25] and led to a reduced proportion of the circulating immature CD56^bright^ NK cells and an increased proportion of the CD56^dim^ NK cell fraction (Fig. 2B) [60, 63–65, 68]. Our longitudinal study also confirmed that these effects could be observed already after the first month of treatment and were maintained over 1 year of regular fingolimod intake. The decreased frequency of CD56^bright^ and the increased frequency of CD56^dim^ could be explained by their different forms of recirculation. CD56^bright^ may be more susceptible to sequestration within secondary lymphoid tissue (SLT) and CD56^dim^ to exit SLT, because contrary to the CD56^dim^, CD56^bright^ cells express high levels of the homing receptors L-selectin and CCR7 [83, 84] and, in addition, CD56^dim^ cells appear to express high levels of S1P_5_ receptors, that appear to be resistant to fingolimod effects in vivo [24, 60, 63].

Moreover, we analysed additional phenotypic markers in the peripheral blood that included CD16, CD94, or the KIRs (Fig. 3). Highly mature CD56^dim^ NK cells are characterised by low CD94, low NKG2A, and high KIR levels, and by a low IFN-gamma, but a high granzyme B and perforin production [78, 79]. Here, we showed that over 12 months of treatment, the fraction of CD56^dim^CD94^low^ was significantly enriched in the circulation, suggesting not only a predomination of mature CD56^dim^ but rather a continuous shift within the CD56^dim^ population towards fully maturation and differentiation. These findings were also in line with a mild increase in the frequency of mature CD56^dim^NKG2A^−^KIR^+^ after fingolimod initiation.

Our data further revealed that under fingolimod therapy, NK cells showed an altered functional receptor profile, with a lower percentage in expression of CCR7 and CX_3_CR1, the inhibitory receptor NKG2A, as well as the activating receptors NKp46 and DNAM-1 (Fig. 4; supplementary Fig. 1). Effects on CCR7 were already reported in treated patients versus untreated MS patients and healthy donors [60]. CD56^bright^ NK cells are up to 60% positive for CCR7 [83] and represents around 95% of the lymph node NK cells [85], while only 5% of CD56^dim^ NK cells are CCR7^+^ [83]. Our data also confirmed that around 5% of the CD56^dim^ express CCR7 at baseline, and that this small population, with probably an intermediary mature phenotype, seems to be also sequestered within the lymph nodes during treatment.

Further, we observed that the fraction of CX_3_CR1^+^ NK cells is also diminished during treatment (supplementary Fig. 1). We previously showed that CX_3_CR1 is expressed on mature NK cells [86] and that MS patients have a reduced proportion of circulating CX_3_CR1^+^ NK cells, although increased frequency of peripheral CX_3_CR1^+^ NK cells correlates with an enhanced MS activity [40]. Since most of the patients of our cohort were stable during the study, the decreasing CX_3_CR1 levers might reflect the therapy success.

We also measured a decrease in the frequency of NKp46 and DNAM-1 in peripheral blood. DNAM-1 decrease in fingolimod treated patients might result in a reduced anti-tumour capacity. In MS patients, a reduced ability to kill activated CD4^+^ T-cells via activation of DNAM-1 is described [41]. DNAM-1^+^ NK cells enriched within the SLT may kill even more efficiently those autoreactive T cells [41]. In the context of our study, the decrease of DNAM-1 in the peripheral blood could be explained by the shift of NK cells towards a fully differentiated [87] or even exhausted [88] profile. Both markers, DNAM-1 and NKp46, play an important role in the innate immune response against human cytomegalovirus infected dendritic cells; NKp46 is furthermore involved in recognition and defence of several other viruses [89–92]. A relative reduction of these receptors in the peripheral blood might lead to a higher susceptibility of fingolimod treated patients for viral infections.

Further, using the flow cytometry data combined with an unsupervised clustering, we identified 15 NK cell clusters that changed in frequency during fingolimod therapy and compared these alterations in responders and non-responders. Although the sample size is very limited, we decided to conduct an exploratory analysis based on response to provide a basis for comparison with studies that reported on associations between certain NK cell subtype and clinical outcome. We confirmed a significant reduction in the ratio of CD56^bright^ clusters and an alteration in two specific CD56^dim^ NK cell clusters (MC4, 11) (Fig. 5). During treatment only the fraction of MC4 increased, while MC11 decreased significantly (Fig. 6A). Compared to MC11, MC4 is characterized by a low expression of CD56, CD94, NKG2A, and NKG2D, but a higher expression of CD16 and KIR. These results indicate that MC4 may represent a fully mature or even exhausted cluster predominant in fingolimod treated patients, while MC11 represents an intermediate mature MC, which decreased with the treatment.

In our GEE analysis, no correlation was observed between MC4 and treatment response or clinical outcomes. However, we did observe an association between an increased frequency of CD56^dim^CD94^low^ (characteristic for MC4 and MC8) and a lower number of infections. CD56^dim^CD94^low^ cells are cells with great capacities to produce intracellular perforin and granzyme B [78]. In the same line, decreased CD56^bright^ and CD56^dim^CD94^low^ frequencies were inversely associated with an increased number of relapses. These results are certainly interesting but need further validation due to small patient numbers in our study.

In our cluster analysis, CD56^bright^ frequency was higher at baseline in patients considered as non-responders compared to those considered as responders. However, after fingolimod-treatment, frequencies of CD56^bright^ and CD56^dim^ were comparable in both groups; thus, the decrease in CD56^bright^ MCs and accordingly the increase in CD56^dim^ fraction were more pronounced in the non-responder patients (Fig. 6). Although these data are preliminary due to the limited sample size, they may indicate that a strong treatment-related reduction of the CD56^bright^ cell fraction is associated with the lack of response. In fingolimod treated patients, high frequency of the CD56^bright^ fraction has been associated with stable magnetic resonance imaging [64] and also with response, using an achieved NEDA(no evidence of disease activity)-3 and NEDA-4 status as response criteria [65]. An association between CD56^bright^ cells and reduced relapse rate has been also observed in MS patients during late pregnancy [93]; and in patients treated with daclizumab, CD56^bright^ increase predicted a reduced number of gadolinium enhancing lesions [54]. Thus, our data may also suggest that an increased fraction of circulation CD56^bright^ could be associated with treatment benefits in MS [64]. CD56^bright^ NK cells that are not reaching the blood are probably captured in the SLT [63]. Accumulation of CD56^bright^ in the SLT could facilitate interactions with T cells and dendritic cells [94, 95]. Moreover, the high IL-7 expression within the SLT may promote NK cell surveillance [96]. It is therefore conceivable that CD56^bright^ can be safely stored within SLT and regulate locally adaptive autoimmune response, or exit the SLT strengthened when needed in the periphery. The latter possibly happens in patients who benefit from fingolimod treatment, as they show a lower decrease of CD56^bright^ cells in the peripheral blood during treatment [64]. Furthermore, SLT is described as a compartment of NK cell maturation [97, 98]. Thus, the arrest of NK cells in SLT might lead to further maturation of these cells.

Our data suggest an increased frequency of fully differentiated or even “exhausted” long-lived NK cells in the circulation of fingolimod-treated MS patients in the peripheral blood. Interestingly, although the limited amount of patient’s material did not allow to perform functional tests in vitro, the profile of this NK cell cluster predominant in fingolimod treated patients resembles the phenotype described in elderly healthy people. With age, CD56^dim^ NK cell fraction and the expression of KIR increase [99–102], while the CD56^bright^ fraction and expression of NKG2A and CD94 decrease [101, 103]. In addition, and also in line with our results, a reduction in NKp46 and DNAM-1 has also been observed during age [100, 102, 104], while CD16 expression remains unchanged [103, 105]. This phenotype reflects a NK cell subgroup with diminished NK cytotoxicity that may underline the high risk of infections observed in fingolimod-treated patients and also in elderly people [102, 106, 107]. Furthermore, it is imaginable that an enhanced fraction of “exhausted” CD56^dim^ NK cells may be accompanied by a diminished proportion of detrimental subtypes, contributing to an overall benefit for MS. However, further studies are needed to verify this hypothesis.

The small number of patients included in our study represents an important limitation, which has an impact on the power of our calculations, the balance on the selection of participants, and the detection of small but statistically significant changes. Therefore, the conducted statistic should be understood as exploratory data analysis, and therefore, all p-values should be considered as exploratory ones. Moreover, our study design does not permit to establish causal relationships between changes affecting NK cells and the treatment, since those changes could just reflect the natural MS progression. Another important limitation is that the study lacks an untreated MS control cohort, which, due to ethical reasons, was unfeasible in a 12-months longitudinal study. However, confirming our estimations, other studies comparing effects with cohorts of untreated patients or healthy controls did present similar NK cell alterations exclusively in fingolimod treated patients [60, 61, 63, 65]. On the other side, due to the longitudinal character of our study, samples were frozen after the different visits to permit the simultaneous analysis of all time points of each single patient. This may have advantages in reducing experimental errors, but has the disadvantage that absolute cell accounts cannot be determined. Changes in population’s frequency could be due to redistribution rather than depletion or emergence of particular subpopulations.

In conclusion, our study indicates that fingolimod not only affects the balance between CD56^dim^ and CD56^bright^ NK cells, but also seems to promote a shift to elderly NK cell clusters, which are probably less functional. Along with the well-described effects of fingolimod on the adaptive immune response, this impact on the innate response may support the increased risk of tumour development [108] and/or infections observed in fingolimod-treated patients. No association between a certain NK cell subtype and the treatment response could be demonstrated in this pilot study. Nevertheless, our data suggests an association between the frequency of cytotoxic CD56^dim^CD94^low^ NK cells and lower infection risk.

## Supplementary Information

Below is the link to the electronic supplementary material.

Supplementary file1 (TIF 426 kb)

Supplementary file2 (TIF 471 kb)

Supplementary file3 (TIF 478 kb)

Supplementary file4 (TIF 466 kb)

Supplementary file5 (TIF 454 kb)

Supplementary file6 (TIF 477 kb)

Supplementary file7 (TIF 481 kb)

Supplementary file8 (DOCX 15 kb)

Supplementary file9 (DOCX 14 kb)

Supplementary file10 (PDF 1225 kb)

Supplementary file11 (PDF 1225 kb)

Supplementary file12 (PDF 1225 kb)

Supplementary file13 (PDF 1224 kb)

Supplementary file14 (PDF 2319 kb)

Supplementary file15 (PDF 1224 kb)

Supplementary file16 (PDF 1224 kb)

Supplementary file17 (PDF 1224 kb)

Supplementary file18 (PDF 1224 kb)

Supplementary file19 (PDF 417 kb)

Supplementary file20 (PDF 399 kb)
