# Supplementary figures and images for: Fingolimod Therapy in Multiple Sclerosis Leads to the Enrichment of a Subpopulation of Aged NK Cells
Source: Neurotherapeutics. 2021 Jul 9;18(3):1783–97. doi: 10.1007/s13311-021-01078-7 (PMC8608997; doi:10.1007/s13311-021-01078-7)

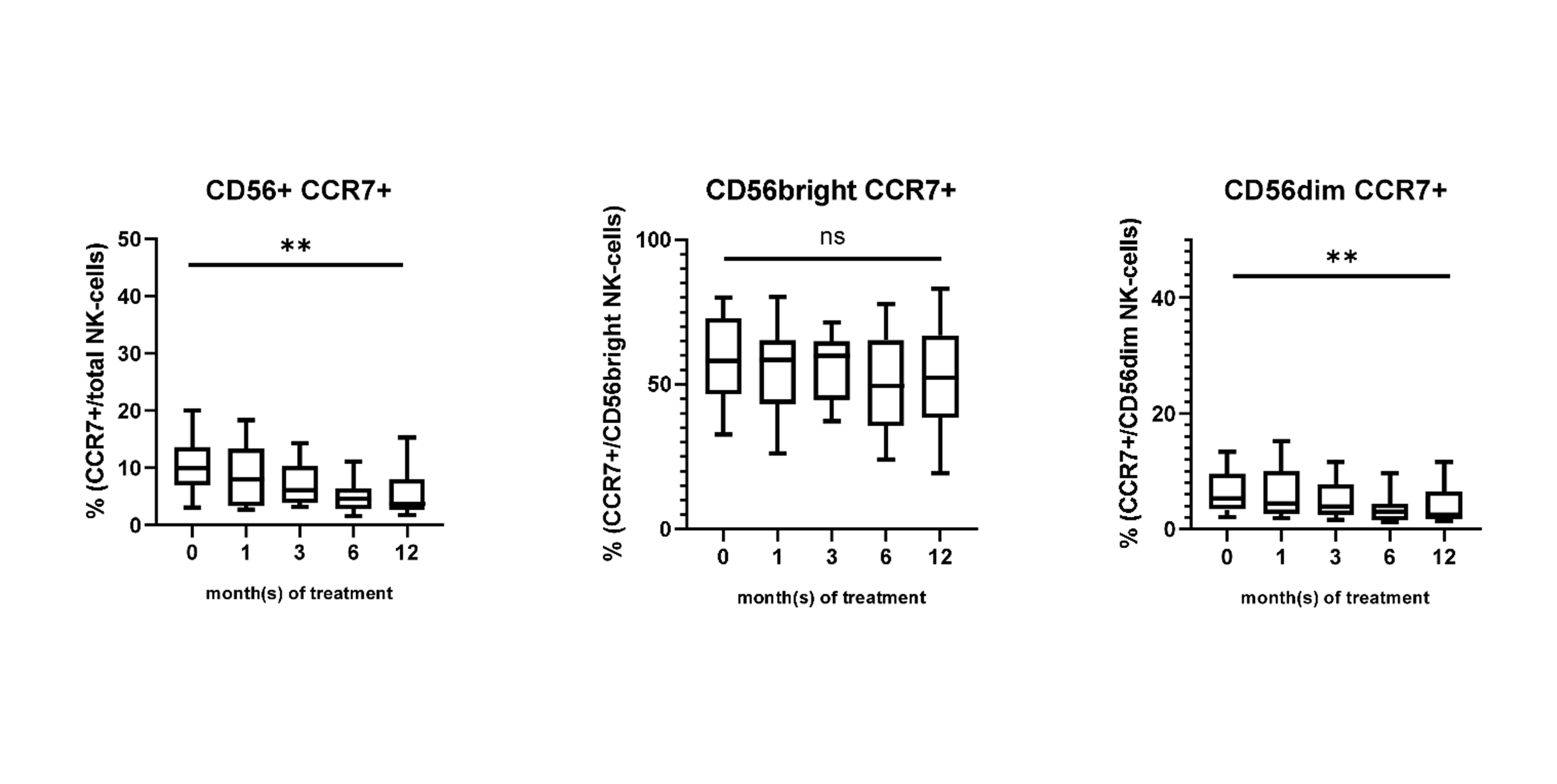

Supplement: Supplementary file 1 — Supplementary file1 (TIF 426 kb) [file 13311_2021_1078_MOESM1_ESM.tif]

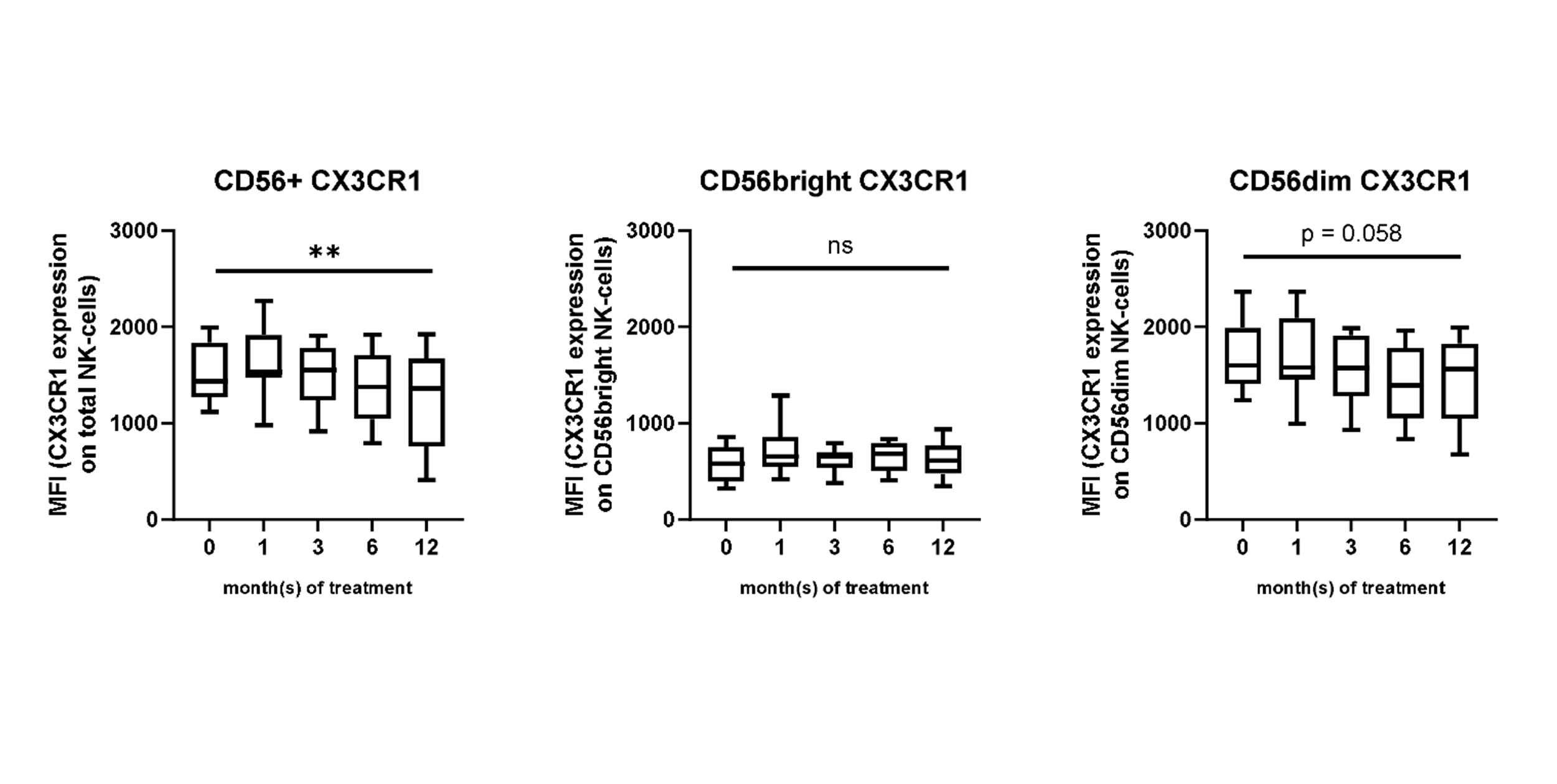

Supplement: Supplementary file 2 — Supplementary file2 (TIF 471 kb) [file 13311_2021_1078_MOESM2_ESM.tif]

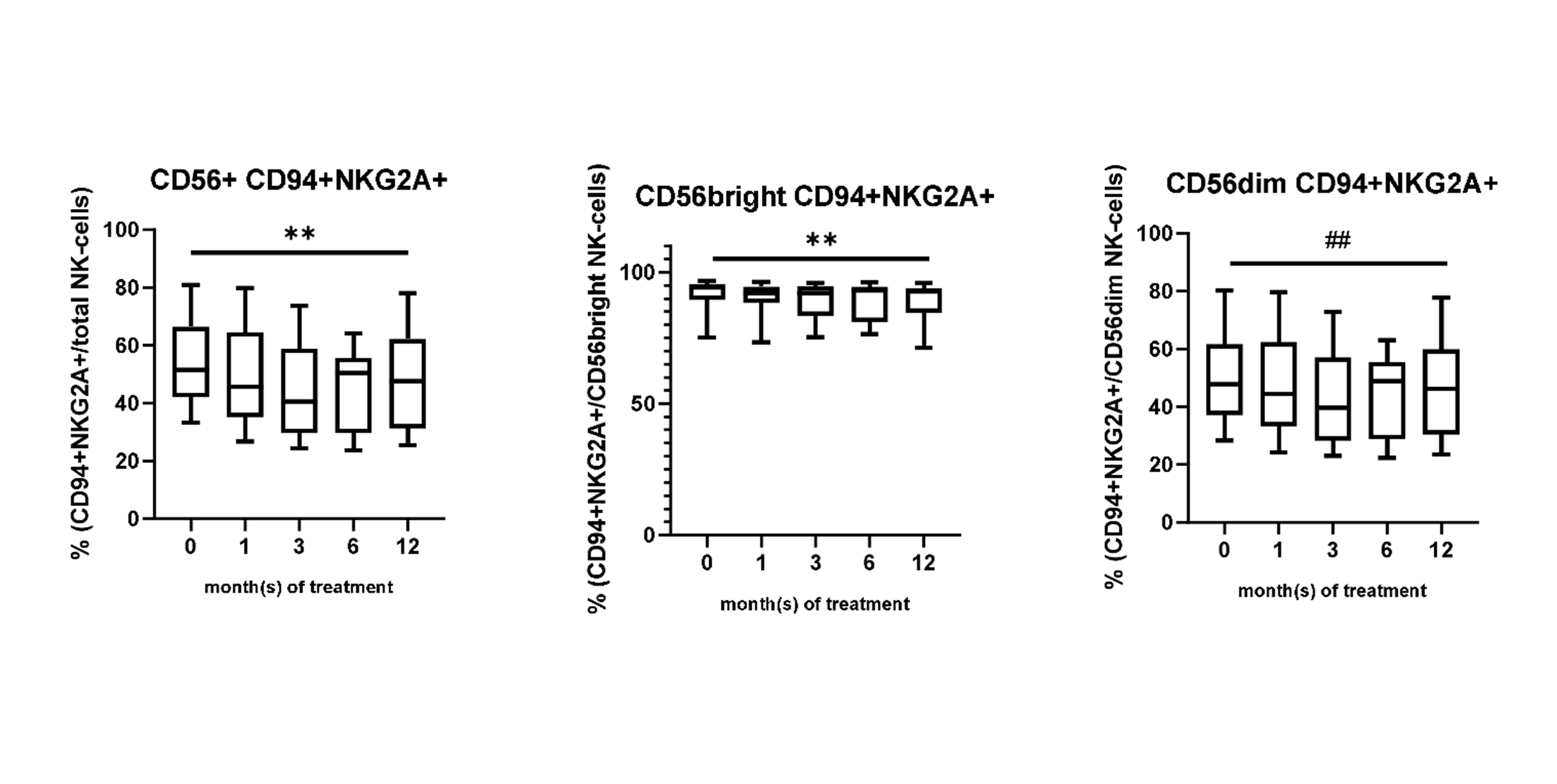

Supplement: Supplementary file 3 — Supplementary file3 (TIF 478 kb) [file 13311_2021_1078_MOESM3_ESM.tif]

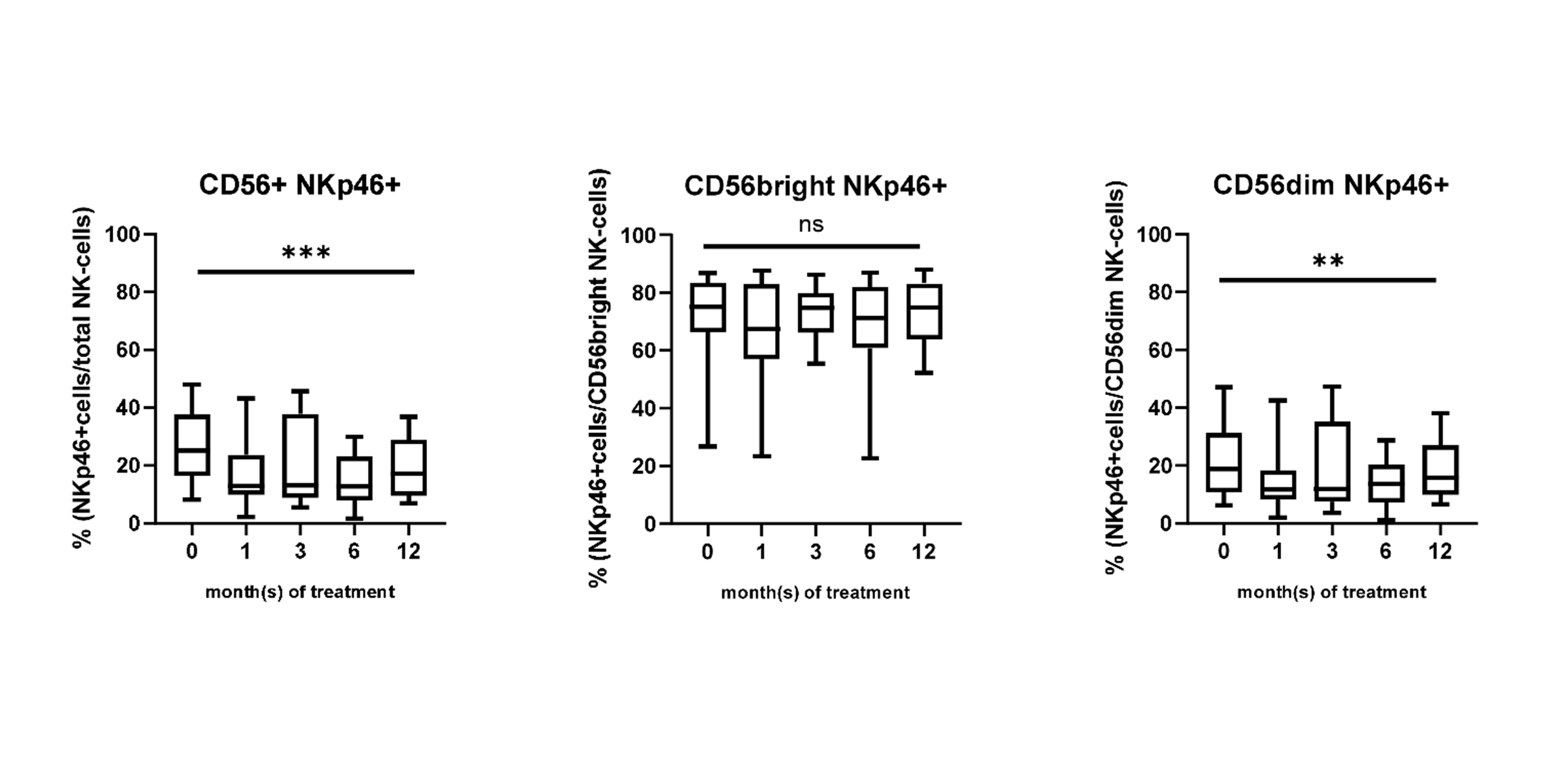

Supplement: Supplementary file 4 — Supplementary file4 (TIF 466 kb) [file 13311_2021_1078_MOESM4_ESM.tif]

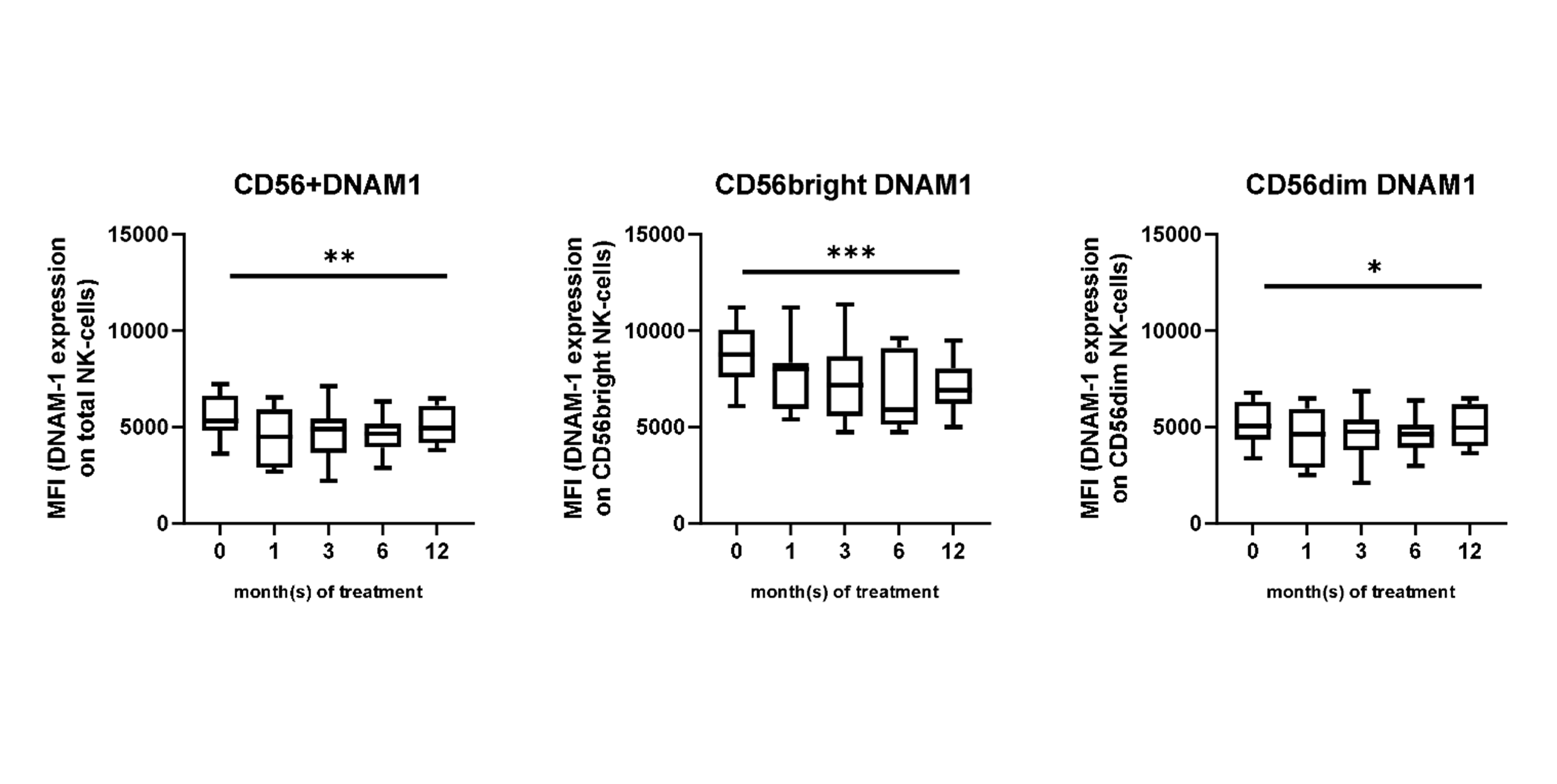

Supplement: Supplementary file 5 — Supplementary file5 (TIF 454 kb) [file 13311_2021_1078_MOESM5_ESM.tif]

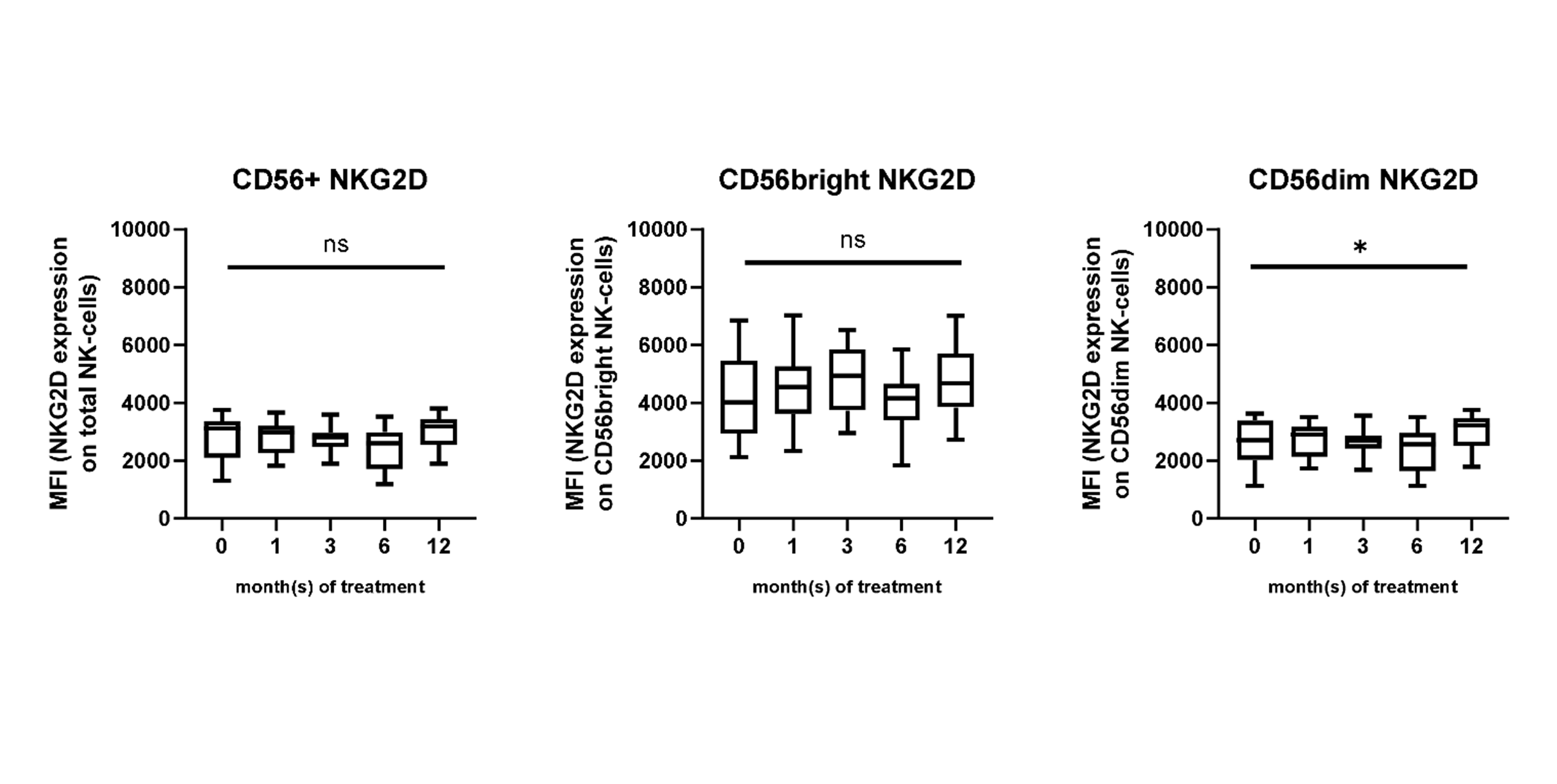

Supplement: Supplementary file 6 — Supplementary file6 (TIF 477 kb) [file 13311_2021_1078_MOESM6_ESM.tif]

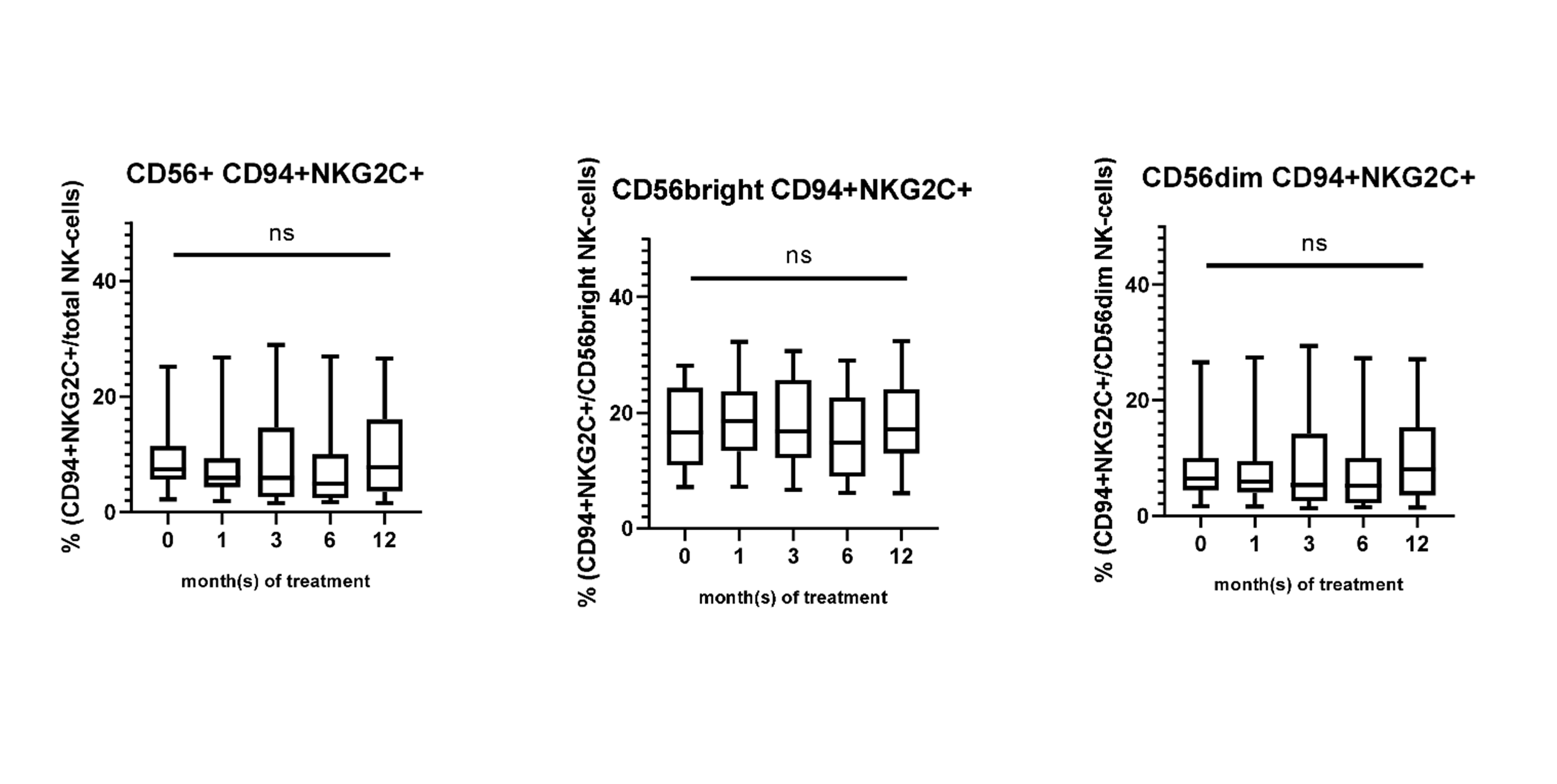

Supplement: Supplementary file 7 — Supplementary file7 (TIF 481 kb) [file 13311_2021_1078_MOESM7_ESM.tif]
