# Supplementary material for: Fingolimod Therapy in Multiple Sclerosis Leads to the Enrichment of a Subpopulation of Aged NK Cells
Source: Neurotherapeutics. 2021 Jul 9;18(3):1783–97. doi: 10.1007/s13311-021-01078-7 (PMC8608997; doi:10.1007/s13311-021-01078-7)
Supplement: Supplementary file 8 — Supplementary file8 (DOCX 15 kb) [file 13311_2021_1078_MOESM8_ESM.docx]

| **Inclusion criteria** | **Exclusion criteria** |
| --- | --- |
| - Definite diagnosis of RRMS according to the 2010 revised McDonald criteria [72] - 18 to 64 years old - Indication for on-label treatment with fingolimod (Gilenya®) according to the current approval - EDSS score ≤ 6.0 - Neurological stable with no evidence of relapse or corticosteroid   treatment within 30 days prior to screening   - Ability to provide written informed consent - Highly effective contraception (Pearl Index < 1), reliable abstinence from any heterosexual relationships, or sterilization of the only partner in women of childbearing potential - Negative pregnancy test at screening and baseline in women of childbearing potential | - Patients with MS manifestations other than RRMS - Patients with known contraindications to fingolimod, in particular: immunodeficiency syndrome, increased risk of opportunistic infections or severe active or chronic active infections (hepatitis, tuberculosis) - History or presence of malignancy (other than localized basal or squamous cell carcinoma of the skin). Severe liver dysfunction (Child Pugh C) - Hypersensitivity against active or any other compound of study medication - 2^nd^ degree Mobitz Type II or higher degree AV block, Sicksinus syndrome, or Sinu-atrial heart block, Significant QT prolongation (QTc>470 msec (female) or >450 msec (males)) - History of symptomatic bradycardia or recurrent syncope, known ischaemic heart disease, cerebrovascular disease, history of myocardial infarction, hypokalaemia, congestive heart failure, history of cardiac arrest, uncontrolled hypertension, or severe sleep apnea. - Patients with clinically significant liver, kidney or bone marrow dysfunction at the time of screening (HB <8.5 g / dl; WBC <2.5 / nl; platelets <100/nl; creatinine clearance by Cockroft-Gault formula: Cl <110ml/min (men) and Cl <95ml/min (women), from age of 30 limit drops 10ml/min per decade; AST / ALT> 3.5 times higher than the upper reference value; bilirubine> 2.0 mg / dl) - Patients without a history of varicella or without vaccination against varicella zoster virus (VZV) and VZV negative antibody serology - Pregnancy or lactation - Participation in another interventional clinical trial within the last 3 months prior to baseline or during the study period - Treatment with natalizumab within the last 3 months prior to baseline, treatment with mitoxantrone, azathioprine or any other immunosuppressive drugs except prednisolone within the last 6 months prior to baseline. - Patients receiving antiarrythmics class Ia (e.g. quinidine, disopyramide) or class III (e.g. amiodarone, sotalol) or beta blockers. Patients receiving heart rate lowering calcium channel blockers (e.g. verapamil, diltiazem or ivabradine) or other substances which may decrease heart rate (e.g. digoxin, anticholinesteratic agents or pilocarpine). - Medical, psychiatric or other conditions that limit the patient’ s ability to understand the patient’s information, to give informed consent or to follow the study protocol - Lack of consent to the storage and analysis of pseudonymous data in the context of the clinical trial - Prisoners or patients that are housed in a judicial institution |
