# Supplementary material for: Fingolimod Therapy in Multiple Sclerosis Leads to the Enrichment of a Subpopulation of Aged NK Cells
Source: Neurotherapeutics. 2021 Jul 9;18(3):1783–97. doi: 10.1007/s13311-021-01078-7 (PMC8608997; doi:10.1007/s13311-021-01078-7)
Supplement: Supplementary file 9 — Supplementary file9 (DOCX 14 kb) [file 13311_2021_1078_MOESM9_ESM.docx]

|  | **CD56^+^** | | **CD56^bright^** | | **CD56^dim^** | |
| --- | --- | --- | --- | --- | --- | --- |
|  | effect | p-value | effect | p-value | effect | p-value |
| **maturation** |  |  |  |  |  |  |
| CD16 | – | 0.108 | **↑** | **0.049** | – | 0.542 |
| CD94 | **↓** | **0.011** | – | 0.203 | ↓ | 0.058 |
| CD94^+^NKG2A | **↓** | **0.004** | **↓** | **0.002** | **↓** | **0.013** |
| KIR | **↑** | **0.007** | **↑** | **0.091** | **↑** | **0.019** |
| **Chemokine receptors** |  |  |  |  |  |  |
| CCR7 | **↓** | **0.002** | – | 0.155 | **↓** | **0.007** |
| CX_3_CR1 | **↓** | **0.031** | – | 0.168 | ↓ | 0.058 |
| **Inhibition Marker** |  |  |  |  |  |  |
| CD94^+^NKG2A | **↓** | **0.004** | **↓** | **0.002** | **↓** | **0.013** |
| **Activation Markers** |  |  |  |  |  |  |
| NKp46 | **↓** | **<0.0001** | – | 0.561 | **↓** | **0.007** |
| DNAM-1 | **↓** | **0.005** | **↓** | **0.001** | **↓** | **0.035** |
| NKG2D | – | 0.413 | ↑ | 0.067 | **↑** | **0.042** |
| CD94^+^NKG2C | – | 0.531 | – | 0.122 | – | 0.333 |
